# Role of transarterial chemoembolization in relation with sorafenib for patients with advanced hepatocellular carcinoma

**DOI:** 10.18632/oncotarget.11030

**Published:** 2016-08-02

**Authors:** Yeonjung Ha, Danbi Lee, Ju Hyun Shim, Young-Suk Lim, Han Chu Lee, Young-Hwa Chung, Yung Sang Lee, Sook Ryun Park, Min-Hee Ryu, Baek-Yeol Ryoo, Yoon-Koo Kang, Kang Mo Kim

**Affiliations:** ^1^ Department of Gastroenterology, Asan Liver Center, Asan Medical Center, University of Ulsan College of Medicine, Republic of Korea; ^2^ Department of Gastroenterology, CHA Bundang Medical Center, CHA University, Republic of Korea; ^3^ Department of Oncology, Asan Liver Center, Asan Medical Center, University of Ulsan College of Medicine, Republic of Korea

**Keywords:** hepatocellular carcinoma, portal vein invasion, sorafenib, overall survival, transarterial chemoembolization

## Abstract

**Background:**

Although sorafenib is considered standard therapy for advanced hepatocellular carcinoma (HCC), actual treatments vary. We evaluated the effects of different treatment strategies on overall survival.

**Methods:**

A retrospective study of sorafenib-treated patients with advanced HCC was conducted. The primary outcome was overall survival. Prognostic factors were analyzed using multivariate Cox-proportional hazards model.

**Results:**

A total of 658 patients (mean age, 54.5 years; 83.3% male) were analyzed; 293, 129, and 236 patients were treated with sorafenib, a combination therapy of sorafenib and transarterial chemoembolization (TACE), and TACE followed by sorafenib, respectively. Overall, 51.2% of patients treated under the combination strategy had portal vein invasion, whereas 89.9% of patients receiving sorafenib monotherapy had distant metastasis. Median overall survival durations were comparable (11.8 months for sorafenib, 16.2 months for the combination therapy, and 13.5 months for TACE followed by sorafenib; *P* = 0.13). However, among portal vein invasion cases, combination (25.7 months, *P* = 0.002) and TACE followed by sorafenib (14.0 months, *P* = 0.030) treatments were associated with longer overall survival duration compared with than sorafenib monotherapy (5.5 months). In a multivariate model, sorafenib duration (hazard ratio [HR], 0.96, *P* < 0.001) and TACE (HR, 0.24, *P* < 0.001) along with Child-Pugh stage (HR, 1.83, *P* = 0.005) were associated with better survival.

**Conclusions:**

In patients with portal vein invasion, TACE performed concurrently with or before sorafenib administration is associated with better survival.

## INTRODUCTION

Sorafenib is an effective and safe treatment for patients with advanced hepatocellular carcinoma (HCC) and varying degrees of liver dysfunction [1]. However, many methods of patients management were devised prior to the introduction of sorafenib; therefore, treatment strategies still vary considerably among geographical regions and individual physicians.

In the Asia-Pacific region, transarterial chemoembolization (TACE) combined with radiation therapy has traditionally been applied to patients with portal vein invasion [2]. Even after the introduction of sorafenib, TACE remains widely used, either concomitantly with sorafenib or after sorafenib failure. In addition, some physicians choose to continue performing TACE for local tumor control, even in patients who subsequently develop vascular invasion and/or distant metastasis, as long as those lesions are considered clinically insignificant with respect to progression.

A significant number of studies have reported beneficial survival effects of TACE in patients with advanced HCC [3-6]. More recently, some physicians have used sorafenib in combination with TACE in patients with portal vein invasion and/or distant metastasis, although earlier combination therapy studies failed to show consistent results [7-9]. Prospective trials evaluating the treatment outcomes of patients with unresectable HCC have shown that combined approaches are associated with better survival and/or slower progression [10, 11]. However, the results of these studies are not fully generalizable because they only compared effects of TACE with those of conservative care or included a heterogeneous population (Barcelona Clinic Liver Cancer [BCLC] stages B and C together). Accordingly, these reports have several limitations with respect to the lack of a proper control group, a heterogeneous study population, and a small sample size.

To address this evidence gap and attempt to better define the role of TACE in patients with advanced HCC, we performed a retrospective analysis of a large historical cohort to compare the effectiveness of sorafenib with that of other treatment strategies commonly used in clinical practice.

## RESULTS

### Patients

**Figure 1:**
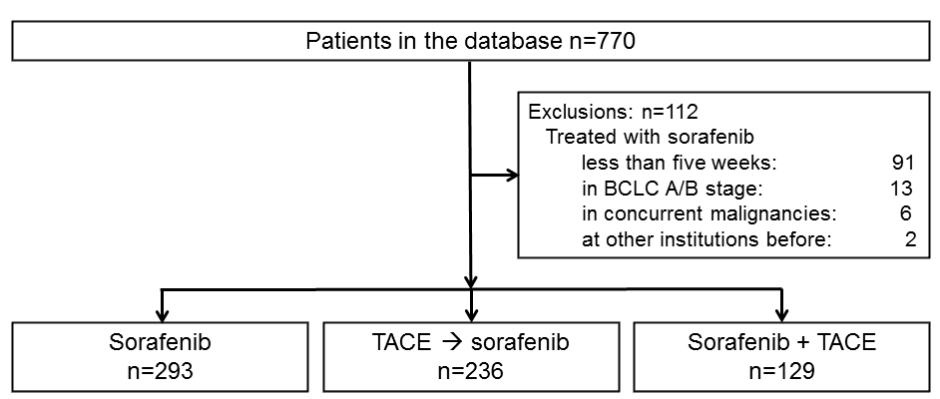
Flow diagram of patient enrollment and distribution into treatment groups

The study flowchart is outlined in Figure 1. Of the 770 patients initially identified, 91 were treated for less than 5 weeks, 13 had BCLC stage A or B disease, 6 had concurrent malignancies at the time of sorafenib treatment, and 2 had already been treated with sorafenib at other institutions. These patients were excluded from the study. The remaining 658 patients were divided according to treatment strategy: 293 patients (44.5%) received sorafenib monotherapy, 129 patients (19.6%) received combination therapy with sorafenib and TACE, and the remaining 236 patients (35.9%) began or continued to receive TACE regardless of vascular invasion, metastasis, and/or poor performance status. Palliative radiation therapy was applied to patients who developed vascular invasion (42.1% of all patients with vascular invasion) and/or metastasis (*data not shown*). Decisions regarding each treatment plan were made by the multidisciplinary tumor board of our center, which comprises experts in hepatology, oncology, interventional radiology, and radiation oncology. This board makes final decisions on the treatment plans of every new, treatment-naïve patients. For patients already receiving therapy, the board held discussions regarding whether treatment strategies should be reconsidered or changed according to the objective clinical. For example, TACE was not generally advised for patients with large infiltrative tumors ( > 50% of total liver volume), extensive portal vein thrombosis, or other medical conditions such as renal insufficiency.

The baseline characteristics of the enrolled patients are presented in Table 1. The study population was predominantly male, with an average age of 54.5 years; 96.3% had tumor-node-metastasis (TNM) stage IV disease. Most patients (86.6%) were hepatitis B virus positive. Portal vein invasion was more common among patients treated with TACE combined with sorafenib (51.2% *vs*. 39.1%), whereas distant metastasis was more common in the sorafenib monotherapy group (89.9% *vs*. 78.3%). More than half of the patients had been previously treated with more than one modality; of these, the most common one was TACE (52.4%). In particular, before progression to BCLC stage C, most patients in the combination (79.8%) and TACE followed by sorafenib (92.8%) groups had been formerly treated with TACE; in contrast, only a small proportion of patients in the sorafenib monotherapy (8.5%) had previously undergone TACE. The three treatment groups were similar with respect to Child-Pugh stage, baseline alpha-fetoprotein (AFP) level, and Eastern Cooperative Oncology Group (ECOG) performance status.

**Table 1:** Demographic data of the study population after stratification by treatment strategy*

	Total (*n* = 658)	Sorafenib (*n* = 293)	TACE followed by sorafenib (*n* = 236)	TACE combined with sorafenib (*n* = 129)	*P* value
Age (years)	54.5 ± 11.0	55.9 ± 11.1	52.9 ± 11.1	54.1 ± 10.1	0.07
Sex [*n* (%)]					
Male	548 (83.3)	239 (81.6)	199 (84.3)	110 (85.3)	0.67
Female	110 (16.7)	54 (18.4)	37 (15.7)	19 (14.7)	
Etiology [*n* (%)]					
Hepatitis B virus	570 (86.6)	257 (87.7)	210 (89.0)	103 (79.9)	
Hepatitis C virus	30 (4.6)	17 (5.8)	5 (2.1)	8 (6.2)	0.19
Alcohol	11 (1.7)	4 (1.7)	4 (1.7)	3 (2.3)	
Others or unknown	47 (7.1)	15 (4.8)	17 (7.2)	15 (11.6)	
Child-Pugh stage [*n* (%)]†					
A	479 (77.1)	209 (80.7)	179 (76.5)	91 (71.1)	0.30
B	142 (22.9)	50 (19.3)	55 (23.5)	37 (28.9)	
C	0 (0.0)	0 (0.0)	0 (0.0)	0 (0.0)	
AFP (ng/mL)	32802.4 ± 156191.9	35497.7 ± 174979.0	38546.8 ± 161986.3	15067.2 ± 78483.0	0.62
Tumor type					0.10
Nodular	331 (50.3)	129 (44.0)	133 (56.4)	69 (53.5)	
Infiltrative	327 (49.7)	164 (56.0)	103 (43.6)	60 (46.5)	
Tumor number§					0.67
Unifocal	142 (42.9)	54 (41.9)	62 (46.6)	26 (37.7)	
Multifocal	189 (57.1)	75 (58.1)	71 (53.4)	43 (62.3)	
Tumor size¶	5.7 ± 4.0	5.7 ± 4.3	5.7 ± 4.1	5.5 ± 3.2	0.98
TNM stage					
I	0 (0.0)	0 (0.0)	0 (0.0)	0 (0.0)	
II	5 (0.8)	2 (0.7)	2 (0.9)	1 (0.8)	0.60
III	19 (2.9)	9 (3.0)	8 (3.3)	2 (1.5)	
IV	634 (96.3)	282 (96.2)	226 (95.8)	126 (97.7)	
ECOG performance status					
0	426 (64.7)	190 (64.8)	143 (60.6)	93 (72.1)	0.25
1	232 (35.3)	103 (35.2)	93 (39.4)	36 (27.9)	
Portal vein invasion [*n* (%)]	257 (39.1)	75 (25.6)	116 (49.2)	66 (51.2)	<0.001
Distant metastasis [*n* (%)]	515 (78.3)	266 (89.9)	145 (61.4)	104 (80.6)	<0.001
Prior therapy [*n* (%)]‡					
TACE	345 (52.4)	25 (8.5)	219 (92.8)	103 (79.8)	<0.001
Local ablation	25 (3.8)	10 (3.4)	10 (4.2)	6 (4.7)	0.88
Resection	51 (7.8)	21 (7.2)	23 (9.7)	6 (4.7)	0.43

* Data are presented as means ± standard deviations.

† 37 missing values.

§ Tumor numbers could not be calculated in patients with infiltrative disease.

¶ Tumor size was calculated by summing the diameters of the two largest target lesions. In patients with infiltrative disease, the tumor size could not be measured.

‡ Before diagnosis of BCLC stage C HCC.

AFP, alpha-fetoprotein; BCLC, Barcelona Clinic Liver Cancer; TNM, tumor-node-metastasis; TACE, transarterial chemoembolization

### Sorafenib administration

In 482 (73.3%) patients, sorafenib therapy was initiated at a standard daily dose of 800 mg. The remaining 176 (26.7%) patients initially received a daily dose of 400 mg because of impaired liver function and/or cytopenia related to portal hypertension. During sorafenib treatment, 97 (14.7%) of all patients experienced grade ≥2 drug-related adverse reactions that lead to dose modification. The total duration of sorafenib treatment was significantly shorter in patients that switched from TACE to sorafenib (mean, 82.0 days; standard deviation [SD], 89.3 days; *P* = 0.002, see Table 1, Supplemental Material). In this group, sorafenib was initiated after an average of 227.7 days (SD, 212.2 days), and multiple TACE sessions (mean, 4.4 sessions; SD, 3.4 sessions) were performed during this period.

The average daily dose of sorafenib was comparable among the groups. Regarding liver function, which was classified roughly according to Child-Pugh stage, no difference in daily sorafenib dose was observed between patients with Child-Pugh A and B liver functions. However, the total treatment duration was shorter in the Child-Pugh B group (mean, 141 days *vs*. 85 days, *P* = 0.038).

Of 658 patients, 652 eventually stopped sorafenib treatment for various reasons (Table 2), the most common of which was disease progression (64.6%), followed by adverse drug reactions (13.3%), physicians' decisions (3.2%), exacerbated liver function or death (0.9%), and economic issues (0.9%). The withdrawal reason(s) could not be evaluated for 69 (10.6%) patients.

**Table 2:** Reasons of withdrawal from sorafenib*

	Total (*n* = 652)	Sorafenib (*n* = 291)	TACE followed by sorafenib (*n* = 236)	TACE combined with sorafenib (*n* = 125)	*P* value
Disease progression [*n* (%)]	421 (64.6)	193 (66.4)	163 (68.9)	65 (52.3)	0.10
Adverse drug reactions [*n* (%)]	87 (13.3)	38 (13.2)	22 (9.3)	27 (21.5)	0.06
Worsening liver function or death [*n* (%)]	6 (0.9)	4 (1.3)	0 (0.0)	2 (1.5)	0.87
Physicians' decision [*n* (%)]	21 (3.2)	6 (2.0)	6 (2.5)	9 (7.2)	0.08
Economic issue [*n* (%)]	6 (0.9)	0 (0.0)	6 (2.5)	0 (0.0)	0.07
Unknown (including loss of follow-up) [*n* (%)]	69 (10.6)	28 (9.6)	27 (11.5)	14 (10.8)	0.92

* Data are presented as means ± standard deviations.

TACE, transarterial chemoembolization

Regarding adverse drug reactions, 616 sorafenib-treated patients (93.6%) experienced one or more adverse drug reactions (Table 3), including hand-foot skin reaction (43.0%), diarrhea (19.7%), anorexia/nausea/vomiting (10.9%), hypertension (2.9%), and fatigue (2.6%). There were no significant differences in the patterns of adverse reactions between the treatment groups, with the exception of an increased incidence of hypertension in the sorafenib monotherapy group (4.8%; *P* = 0.036). When adverse drug reactions were classified according to Child-Pugh stage, hand-foot skin reactions were more frequent in the Child-Pugh A group (46.8% *vs*. 19.9%, *P* < 0.001), whereas the rates of other adverse reactions were similar between groups.

**Table 3:** Adverse drug reactions in the overall group and across subgroups and Child-Pugh classes*

	Total (*n* = 616)	Sorafenib (*n* = 290)	TACE followed by sorafenib (*n* = 222)	TACE combined with sorafenib (*n* = 104)	*P* value
Hand-foot skin reaction	265 (43.0)	120 (41.4)	101 (45.5)	44 (42.3)	0.46
Diarrhea	121 (19.7)	59 (20.3)	35 (15.8)	27 (26.0)	0.35
Hypertension	18 (2.9)	14 (4.8)	4 (1.8)	0 (0.0)	0.036
Anorexia, nausea, vomiting	67 (10.9)	28 (9.7)	25 (11.3)	14 (13.5)	0.40
Fatigue	16 (2.6)	5 (1.7)	7 (3.2)	4 (3.8)	0.95
Others	129 (20.9)	64 (22.1)	50 (22.5)	15 (14.4)	0.22

	Total (*n* = 621)†	Child-Pugh A (*n* = 479)	Child-Pugh B (*n* = 142)	Child-Pugh C (*n* = 0)	*P* value
Hand-foot skin reaction	248 (39.9)	224 (46.8)	24 (16.9)	-	<0.001
Diarrhea	124 (20.0)	101 (21.1)	23 (16.2)	-	0.39
Hypertension	21 (3.4)	21 (4.4)	0 (0.0)	-	0.12
Anorexia	66 (10.6)	50 (10.4)	16 (11.3)	-	1.00
Fatigue	19 (3.1)	16 (3.3)	3 (2.1)	-	1.00
Others	119 (19.2)	93 (19.4)	26 (18.3)	-	0.86

* Data are presented as means ± standard deviations.

† 37 missing values in the Child-Pugh stage analysis.

TACE, transarterial chemoembolization

### Survival

**Figure 2:**
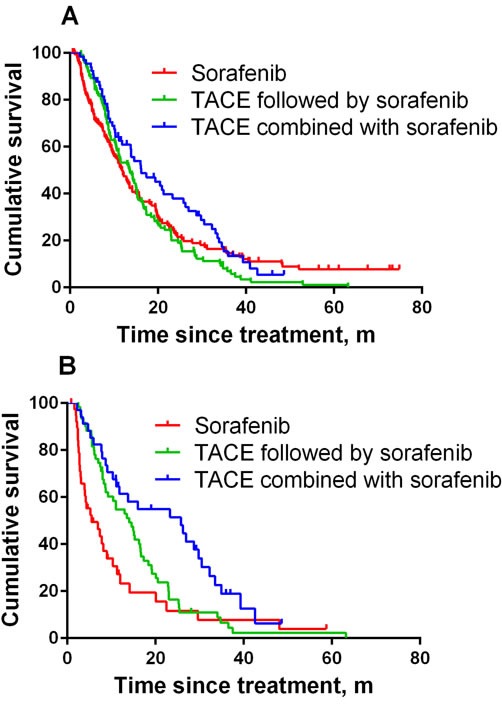
**A.** Graphical representation of the relationship between treatment strategies and overall survival in all subjects. **B.** Graphical representation of the relationship between treatment strategies and overall survival in subjects with portal vein invasion, with or without distant metastasis.

From the time when sorafenib was indicated, patients treated with sorafenib monotherapy had overall survival times comparable to those of patients who received the combination therapy, as well as of patients treated with TACE followed by sorafenib (median, 11.8 [95% confidence interval (CI), 9.7-13.9] months for the sorafenib monotherapy group; 13.5 [10.6-16.3] months for TACE followed by sorafenib group; 16.2 [9.5-22.9] months for the combination therapy group; *P* = 0.13; Figure 2A).

Among patients with portal vein invasion, the survival durations with combination therapy (25.7 [9.7-41.8] months, *P* = 0.002) and TACE followed by sorafenib (14.0 [9.8-18.3] months, *P* = 0.030) were significantly longer than that with sorafenib monotherapy (5.5 [1.7-9.6] months; Figure 2B), regardless of the metastatic status to distant organ(s) at the time of sorafenib indication. Among TACE-treated patients, concomitant therapy was associated with better survival (*P* = 0.011).

Univariate and multivariate associations between treatment outcomes and clinical characteristics among overall patients and those with portal vein invasion are presented in Tables 4 and 5. Among the overall patient group, the overall survival outcome was independently associated with the Child-Pugh stage (hazard ratio [HR], 1.65, 95% CI, 1.06-2.55, *P* = 0.026) and sorafenib treatment duration (per week) (HR, 0.97, 95% CI, 0.95-0.99, *P* < 0.001). However, in the subgroup of patients with portal vein invasion, TACE during the study period was significantly associated with overall survival (HR, 0.24, 95% CI 0.15-0.41, *P* < 0.001), as were the Child-Pugh stage (HR, 1.83, 95% CI, 1.20-2.79, *P* = 0.005) and sorafenib treatment duration (HR, 0.96, 95% CI, 0.94-0.98, *P* < 0.001).

**Table 4:** Hazard ratios of clinical characteristics associated with overall mortality in the overall patient population

Univariate model	HR (95% CI)	*P* value
Age, per 1 year	0.99 (0.98-1.00)	0.031
Sex, male	0.89 (0.65-1.22)	0.46
Etiology		0.87
Hepatitis B virus	1.17 (0.67-2.05)	0.58
Hepatitis C virus	0.84 (0.31-2.26)	0.73
Others or unknown	0.90 (0.58-1.39)	0.62
Child-Pugh stage, B	2.05 (1.54-2.72)	<0.001
AFP ≥ 400 ng/mL	1.46 (1.14-1.86)	0.002
ECOG performance status, 1	1.65 (1.30-2.11)	<0.001
Duration of sorafenib treatment, per 1 week	0.98 (0.97-0.98)	<0.001
TACE procedure during the study period	0.95 (0.75-1.21)	0.70
Presence of portal vein invasion	1.19 (0.94-1.50)	0.16
Radiation therapy for portal vein invasion	0.71 (0.48-1.04)	0.08
Presence of distant metastasis	0.90 (0.68-1.19)	0.44
Multivariate model	OR (95% CI)	*P* value
Age, per 1 year	0.99 (0.97-1.02)	0.56
Child-Pugh stage, B	1.65 (1.06-2.55)	0.026
AFP ≥ 400 ng/mL	1.01 (0.65-1.57)	0.98
ECOG performance status, 1	1.32 (0.88-1.99)	0.18
Duration of sorafenib treatment, per 1 week	0.97 (0.95-0.99)	<0.001
Radiation therapy for portal vein invasion	0.68 (0.44-1.04)	0.07

HR, hazard ratio; CI, confidence interval; AFP, alpha fetoprotein; ECOG, Eastern Cooperative Oncology Group; TACE, transarterial chemoembolization

**Table 5:** Hazard ratios of clinical characteristics associated with overall mortality in patients with portal vein invasion with or without distant metastasis

Univariate model	HR (95% CI)	*P* value
Age, per 1 year	0.99 (0.97-1.01)	0.23
Sex, male	0.87 (0.50-1.50)	0.61
Etiology		0.92
Hepatitis B virus	1.15 (0.42-3.17)	0.79
Hepatitis C virus	1.21 (0.30-4.96)	0.79
Others or unknown	1.21 (0.66-2.22)	0.54
Child-Pugh stage, B	1.59 (1.07-2.37)	0.021
AFP ≥ 400 ng/mL	1.19 (0.80-1.78)	0.39
ECOG performance status, 1	1.87 (1.27-2.76)	0.001
Duration of sorafenib treatment, per 1 week	0.97 (0.95-0.98)	<0.001
TACE procedure during the study period	0.47 (0.30-0.72)	0.001
Radiation therapy for portal vein invasion	0.71 (0.48-1.04)	0.08
Presence of distant metastasis	1.01 (0.69-1.47)	0.97
Multivariate model	OR (95% CI)	*P* value
Child-Pugh stage, B	1.83 (1.20-2.79)	0.005
ECOG performance status, 1	1.37 (0.92-2.04)	0.12
Duration of sorafenib treatment, per 1 week	0.96 (0.94-0.98)	<0.001
TACE procedure during the study period	0.24 (0.15-0.41)	<0.001
Radiation therapy for portal vein invasion	0.79 (0.51-1.25)	0.32

HR, hazard ratio; CI, confidence interval; AFP, alpha fetoprotein; ECOG, Eastern Cooperative Oncology Group; TACE, transarterial chemoembolization

### Post-sorafenib treatments

Post-sorafenib treatment was defined as any treatment attempted after sorafenib cessation. Approximately half of our current study patients (44.4%) received no further treatment after sorafenib cessation (Table 6). Radiation therapy was attempted in 15.2% of all patients. This therapy was mainly applied to metastatic lesions at distant sites and to the main and/or first branches of portal vein thrombosis for patients in the TACE followed by sorafenib group. Other treatments, such as systemic non-sorafenib chemotherapy, were attempted in 27.2% of all patients.

**Table 6:** Treatment(s) administered after sorafenib cessation*

	Total (*n* = 658)	Sorafenib (*n* = 293)	TACE followed by sorafenib (*n* = 236)	TACE combined with sorafenib (*n* = 129)	*P* value
No treatment	292 (44.4)	130 (44.4)	103 (43.6)	59 (45.7)	0.80
Radiation therapy	100 (15.2)	49 (16.7)	37 (15.7)	14 (10.9)	0.87
TACE	87 (13.2)	18 (6.1)	41 (17.4)	28 (21.7)	<0.001
Others	179 (27.2)	96 (32.8)	55 (23.3)	28 (21.7)	0.13

* 79 patients treated with more than one modality after sorafenib cessation. TACE, transarterial chemoembolization

## DISCUSSION

In our present study, we compared the efficacies of various therapeutic options among patients with BCLC stage C HCC. Patients treated with sorafenib monotherapy exhibited overall survival outcomes similar to those of patients undergoing TACE concomitantly with or prior to sorafenib initiation, although the survival duration was the longest in the combination therapy group. However, among patients with portal vein invasion, combination therapy with TACE and sorafenib demonstrated a longer overall survival compared with sorafenib monotherapy. The probable beneficial effect of TACE was again observed in univariate and subsequent multivariate analyses, which demonstrated that TACE during the study period, as well as Child-Pugh liver function status and sorafenib duration, were independent prognostic factors for overall survival.

It could be argued that differences in survival are consequences of different baseline characteristics of patients allocated to each treatment strategy. Patients treated with the combination therapy or TACE followed by sorafenib had a higher rate of portal vein invasion than did those in the sorafenib monotherapy group, whereas patients who received sorafenib monotherapy had a higher rate of distant metastasis. Notably, patients treated with TACE either prior to or in combination with sorafenib had undergone various treatments before being diagnosed with BCLC stage C HCC. In contrast, a majority of sorafenib monotherapy patients (82.3%) had not received any previous treatments. As distant metastasis was the actual trigger for sorafenib initiation in > 80% of our patients (*data not shown*), subjects treated with TACE at earlier stages of disease tended to continue this procedure even upon progression to advanced stage, unless there was distant metastasis. On the other hand, patients newly diagnosed with metastatic HCC were likely to receive sorafenib monotherapy. This phenomenon reflects the tendency of our institution to regard patients with portal vein invasion as candidates for locoregional treatments such as TACE.

Several studies reported the potential benefit of combined treatment with TACE and sorafenib. According to recent studies on patients with advanced HCC, combination therapy with TACE and sorafenib demonstrated better survival than treatment with sorafenib monotherapy [8, 12]. In addition, in a final analysis of the Global Investigation of therapeutic DEcisions in hepatocellular carcinoma and Of its treatment with sorafeNib study, combination therapy was associated with a significantly longer survival duration compared with sorafenib monotherapy in patients with BCLC stage C disease as well as in the overall study population [13]. Several other studies have also reported that patients treated with combination therapy had longer survival duration [14-16]; however, survival outcomes in these studies, in which the study populations comprised partly or almost entirely BCLC stage B patients, were compared with those of TACE monotherapy. Although our study yielded results similar to those of the above-mentioned studies and included a larger number of patients receiving combination therapy, the current meta-analyses found that combination therapy only prolonged the time-to-progression but not overall survival [17, 18]. Therefore, further prospective randomized controlled trials are needed to confirm the clinical benefit of the combination therapy of TACE and sorafenib.

Regarding sorafenib dosing and treatment duration, the patients in our present cohort (all Korean, none with a Child-Pugh C liver function status) received approximately 82.5% of the recommended dosages. Although dosages were similar among patients with different liver function profiles, the total duration was shorter among those who switched from TACE to sorafenib, probably because most patients would have experienced a marked decline in liver function after repeated TACE that would reduce their ability to tolerate another toxic agent. Patients with Child-Pugh stage B HCC who are vulnerable to worsening of liver function also received a significantly shorter course of sorafenib treatment. In addition, economic issues must have affected the treatment duration, as sorafenib treatment for patients with Child-Pugh B liver function is currently not reimbursed by the Korean national health insurance program.

From the perspective of adverse drug reactions, our results are in line with previous studies that reported hand-foot skin reactions and diarrhea as being the most common side effects [1, 19-22]. The incidence of various adverse drug reactions was similar across the treatment groups and most patients were able to continue sorafenib treatment despite the side effects. Rather, a majority of sorafenib-treated patients who ceased sorafenib treatment did so because of disease progression.

Approximately half of our patients who discontinued sorafenib for various reasons received no further treatment regardless of their initial therapeutic strategy. Among the remaining patients, roughly a quarter were treated with other systemic chemotherapies and approximately 15% received radiation therapy for extrahepatic metastasis control. Patients treated with sorafenib either initially or after TACE were less likely to convert to or resume TACE therapy than patients in the combination therapy group who continued to receive TACE even after stopping sorafenib. This finding was not unexpected because patients initially treated with sorafenib had a higher rate of distant metastasis and those who switched to sorafenib after TACE therapy would have already experienced TACE failure; therefore, most such patients were not suitable for locoregional treatment.

Although BCLC stage C encompasses a heterogeneous population, the current BCLC policy recommends the same treatment for all patients. Our findings suggest that TACE could improve the survival of patients with portal vein invasion. Differences in baseline characteristics may have influenced our results. Nonetheless, our study clearly shows that individualized treatment approaches, not solely sorafenib, are being attempted for BCLC stage C HCC patients in actual clinical practice and reveals survival differences among these various approaches. Local intrahepatic lesion control may remain important after the development of vascular invasion. Further prospective studies are needed to determine whether concomitant therapeutic approaches are beneficial in patients with advanced HCC. In addition, the role of TACE in patients with locally advanced disease who experience sorafenib failure should be defined.

Our current study, which included a relatively large number of patients, conducted in-depth survival analyses according to therapeutic strategy, using sorafenib monotherapy as a control. The principal methodological limitation of this study was our retrospective use of medical records for data collection. In addition, the generalizability of our study is somewhat limited because it was performed at a single Asian center and included patients with advanced HCC only if they received sorafenib. Therefore, we cannot conclude definitively that the entire BCLC stage C population should be considered with regard to combination therapy, but rather suggest that some candidates for sorafenib may further benefit from the addition of TACE. Of course, at what point and by which criteria TACE should be stopped or continued in both advanced and intermediate HCC patients still require clarification, and we are currently investigating this issue.

In conclusion, TACE, likely in addition to sorafenib, can prolong survival in HCC patients with portal vein invasion. Well-designed prospective studies are needed to validate this finding.

## MATERIALS AND METHODS

### Study design

Our institutional review board approved this study in accordance with the Declaration of Helsinki of the World Medical Association and waived the requirement to obtain informed consent. The inclusion criteria were as follows: 1) histologic and radiologic diagnosis of BCLC stage C HCC from 2007 to 2010 and 2) sorafenib treatment for at least 5 weeks. Patients who met at least one of the following criteria from 2007 to 2010 were excluded: 1) histologic or radiologic diagnosis of HCC other than BCLC stage C, 2) sorafenib treatment duration of < 5 weeks, 3) concurrent malignancies, or 4) previous sorafenib treatment at other institutions. Patients diagnosed with BCLC stage C HCC before 2007 were excluded from the analysis because sorafenib was not readily available in Korea at that time. There was no limit with regard to the treatment modalities applied prior to 2007, as long as patients had not received treatment for BCLC stage C disease.

The database of our institution contains data on all sorafenib-treated HCC patients, including demographics, liver disease etiologies, tumor characteristics, Child-Pugh stage, AFP level, performance status, radiologic data, and follow-up over a 5-year period. The treatment sequence for each individual patient was identified through a review of the electronic medical records. Responses to treatment were assessed using the Response Evaluation Criteria In Solid Tumors (RECIST) [23, 24] until 2010 and using the modified RECIST [25] beginning in 2010. For all patients in this study, the response evaluation was confirmed using the modified RECIST [25].

### TACE Protocol

TACE was performed according to a decision of the managing physicians that this procedure may improve survival by achieving local tumor control.

The routine standardized TACE protocol used in our hospital was previously described (see text, Supplemental Material) [26].

All TACE sessions were uniformly performed by skilled interventional radiologists with > 5 years of experience. Dynamic contrast-enhanced liver imaging was performed 4-6 weeks after TACE to assess the effect of the procedure. TACE was principally repeated every 6-8 weeks to treat residual tumors, assuming that hepatic function was preserved. The duration of TACE treatment was determined at the attending physician's discretion.

### Sorafenib administration

For the analysis of sorafenib administration, the period between the first and last administrations of sorafenib was considered. The last administration was defined as cessation of the drug for ≥7 consecutive days; temporal cessation of < 7 consecutive days was not considered treatment termination. Principally, sorafenib was initiated at a dose of 400 mg twice daily, and this dose was reduced by 200 mg in the incidence of grade ≥2 drug-related adverse reactions according to the National Cancer Institute Common Terminology Criteria for Adverse Events [27]. The occurrence of any of the following was defined as an adverse reaction: (i) hand-foot skin reaction; (ii) diarrhea; (iii) hypertension; (iv) anorexia, nausea, vomiting; (v) fatigue; and (vi) others. Stepwise dose reescalation by 200 mg was performed when patients had recovered from toxicity and could tolerable the medication.

The average dose and duration of sorafenib treatment, reason(s) for treatment termination, and information about treatment(s) after sorafenib termination were collected from the electronic medical records. The average dose was calculated by dividing the total amount (mg) administered by the total treatment period (days).

### Evaluation of treatment outcomes

Mortality, progression, and follow-up data were collected from the liver patient registry at our institution. The primary outcome measure was overall survival, defined as the elapsed time between the date of sorafenib indication and death or last follow-up. Follow-up was terminated on July 1^st^, 2013 or at an earlier date by censoring for a loss to follow-up. Patients who resumed sorafenib after cessation were also censored at the date on which treatment was restarted.

### Statistical analysis

Frequency tables of patient characteristics, liver disease etiologies, tumor stage, liver function, laboratory data, and performance status were constructed. The Kaplan-Meier method was used to compare the effects of treatment strategies on overall survival, with the sorafenib monotherapy group as a control. We added a subgroup analysis of patients with portal vein invasion. We also performed a univariate analysis to identify prognostic factors which are associated with survival. Factors significantly associated with overall survival in the univariate analysis were entered to a multivariate Cox-proportional hazards regression models. HRs were estimated from the model and are reported with their 95% CIs. All tests for significance were two-tailed, and the level of significance was set at 5%. All analyses were conducted using IBM SPSS version 20.0 software (IBM Corp., Armonk, NY, USA).

## SUPPLEMENTARY MATERIALS TABLE


