# Supplementary material for: Role of transarterial chemoembolization in relation with sorafenib for patients with advanced hepatocellular carcinoma
Source: Oncotarget. 2016 Aug 2;7(45):74303–13. doi: 10.18632/oncotarget.11030 (PMC5342054; doi:10.18632/oncotarget.11030)
Supplement: Supplementary file 1 [file oncotarget-07-74303-s001.pdf]

Role of transarterial chemoembolization in relation with sorafenib for patients with advanced hepatocellular carcinoma

Supplementary Material

TACE

It was performed under local anesthesia with right femoral access. Angiography of the celiac trunk and superior mesenteric artery was initially performed in all patients to evaluate the anatomy, tumor burden, and patency of the portal vein. After angiography, 2 mg/kg cisplatin was administered for 15 min into the right lobar, left lobar, or proper hepatic artery according to the location of the tumor. An emulsion of cisplatin in iodized oil (Lipiodol; Laboratoire Guerbet, Aulnay-Sous-Bois, France) was delivered at a 1:1 ratio, which was followed by embolization with 1 mm diameter absorbable gelatin sponge particles (Gelfoam; Ethicon, Somerville, NJ) in a selective or, if possible, superselective manner until antegrade arterial flow stasis was achieved on angiography. The dose of iodized oil administered depended on the tumor size. Cisplatin injection was preceded by intravenous hydration and antiemetics and followed by further intravenous hydration.

Supplemental Table 1. Dosing and treatment duration of sorafenib, overall and across the subgroups and Child-Pugh stage

	Total (*n*=658)	Sorafenib (*n*=293)	TACE followed by sorafenib (*n*=236)	TACE combined with sorafenib (*n*=129)	*P* value
Daily dosing, mg	661.6 ± 156.5	657.6 ± 157.0	679.8 ± 149.9	636.6 ± 165.5	0.18
Duration, days	126.1 ± 190.5	139.1 ± 227.0	82.0 ± 89.3	178.6 ± 219.4	0.002
	Total (*n*=658)†	Child-Pugh A (*n*=479)	Child-Pugh B (*n*=142)	Child-Pugh C (*n*=0)	
Daily dosing, mg	658.4 ± 156.6	657.3 ± 155.5	662.2 ± 161.5	-	0.82
Duration, days	128.2 ± 199.0	141.1 ± 209.1	84.5 ± 153.3	-	0.038

* Data are presented with mean ± standard deviation. † 37 missing values.

TACE, transarterial chemoembolization
